# Circuit design for broadband decoupling in multi-coil multi-nuclear applications

**DOI:** 10.1016/j.jmr.2025.107924

**Published:** 2025-06-18

**Authors:** Joseph Busher, Edith Touchet-Valle, Jacob Degitz, Mary P. McDougall

**Affiliations:** a Department of Biomedical Engineering, Texas A&M University, 101 Bizzell St., College Station, TX 77843, USA; b Department of Electrical and Computer Engineering, Texas A&M University, 301 Wisenbaker Engineering Building, College Station, TX 77843, USA

**Keywords:** Magnetic resonance imaging, Magnetic resonance spectroscopy, Radiofrequency coils, Multi-nuclear

## Abstract

The wealth of information available from multinuclear magnetic resonance imaging and spectroscopy is largely untapped in the clinical setting. This is due to a multitude of challenges in the pipeline ranging from acquisition strategies, hardware design, processing, and interpretation/analysis. As a small part of addressing these challenges, this work presents a straightforward approach for broadband decoupling between coils. This circuit was created with the implementation of a series PIN diode and was evaluated on the bench and experimentally for ^1^H, ^31^P and ^23^Na at 3 T. Individual coils were single-tuned with this decoupling network and stacked to enable a switched triple-tuned coil. These coils were evaluated in various purposefully coupled configurations and compared to a narrowband trap active detuning network to demonstrate potential modularity of this design. Narrowband trapped coils showed drops in SNR when combined with other coils, presumably due to coupling between receiver elements tuned to different frequencies. This broadband decoupling behavior was shown to be independent of positioning through coils oriented to be nearly perfectly geometrically coupled and the addition of a three-element array of the same size. This configuration was validated on a post-mortem pig to verify the losses of the network did not prohibit its use for preclinical imaging and spectroscopy applications. Although losses were incurred as a result of the broadband decoupling network, it enabled a modular design that can be adapted to a given study without significant compromise of the signal integrity and could eliminate the need for certain custom coils for multi-nuclear studies.

## 1. Introduction

Although not routinely used in the clinic, non-^1^H nuclear magnetic resonance (NMR) can provide biochemical information that further improves the study and evaluation of disease [1]. However, the implementation of X-nucleus magnetic resonance imaging and spectroscopy (MRI/S) is challenging for many reasons. These include any combination of low natural abundance, lower gyromagnetic ratios compared to ^1^H, rapid signal decay, and low sensitivity [1,2]. For this reason, signal excitation and detection hardware, specifically radiofrequency (RF) coils, must be particularly sensitive to address some of these challenges [2,3]. Thus, common techniques in coil design for improving reception sensitivity, such as the implementation of multi-channel phased arrays, become not only more important but also more challenging for multi-nuclear studies due to the behaviors of these circuits at multiple frequencies [4,5].

Several methods of multi-tuning coils have been demonstrated, including the use of traps, switching coils, and nested designs [6–9]. Although multi-tuned circuits are associated with higher losses than their single-tuned counterparts, the need for multi-tuning is required for co-registration of data and to shim within the region of interest [4,10]. Multi-nuclear coil designs can generally be summarized into single structure and multi-structure approaches. Single structure approaches, defined as a single coil conductor tuned to multiple frequencies, may be realized with switching circuitry or with simultaneous multi-tuning designs, such as traps [6,8,11,12]. These approaches enable the development of compact hardware, which is particularly beneficial when designing arrays. However, traps and/or switching circuitry introduce significant resistive losses and add complexity to the system. Multi-structure approaches, on the other hand, are defined as coils with separate conductor geometries for each nucleus [9]. This approach is typically considered to incur less loss relative to single structure approaches since it eliminates the need for complex and lossy multi-tuning circuitry, thus allowing for higher sensitivity; however, this comes at the expense of non-compact designs that complicate implementation into array configurations.

In either case there exists an additional complication, that being the decoupling requirement not only to isolate the Rx coils from transmit (Tx) coils, but also to decouple the multiple Rx coils (which may be operating at different frequencies) from one another. These two decoupling requirements share a common goal of isolating multiple resonant structures, and their approaches largely overlap, so common methods for both decoupling Tx from Rx elements and Rx from Rx elements will be discussed together. Geometric decoupling enables straightforward implementation of a multi-structure approach [13]. However, this decoupling strategy is not feasible in many common instances when it is not possible to achieve orthogonal fields such as quadrature configurations used as array elements or flexible coils [14,15]. Another common approach to minimizing coupling is the insertion of frequency blocking traps [7,16]. Trapping is an effective decoupling option that is independent of the positioning of the coils relative to one another. It is inherently narrowband, however, and the losses incurred with the addition of the traps are dependent on the proximity of the multiple frequencies to one another [17]. This work describes the design and evaluation of a straightforward broadband configuration for decoupling Rx coils from the Tx coil in place of conventional active detuning and has the concurrent benefit of providing switchable broadband decoupling between Rx coils at different frequencies (different nuclei in a multi-structure approach). This design enables modularity as any coil combination is possible without concerns of frequency of operation or position dependent coupling.

This particular work focused on a coil design to enable surface excitation and reception of ^1^H, ^23^Na and ^31^P, in this case for the study of large animal musculoskeletal biomarkers. Two stacked linear butterfly coils were used for excitation to maximize geometric isolation between Tx and Rx coils. Separate loop coils were designed for each nucleus to be stacked enabling a modular triple-tuned coil structure. Broadband decoupling was achieved through the straightforward implementation of a series PIN diode in place of a narrowband active trap for active detuning. This network enabled a broadband network that could be used for both isolating the Rx coils from the Tx coil, but also decoupling Rx coils tuned to different frequencies, enabling multi-nuclear operation. The overall coil system was evaluated using bench measurements, phantom MRI/S studies, and its sensitivity to physiologically relevant biomarkers was preliminarily validated on a post-mortem porcine model.

## 2. Methods

### 2.1. Coil design and construction

**Fig. 1.**
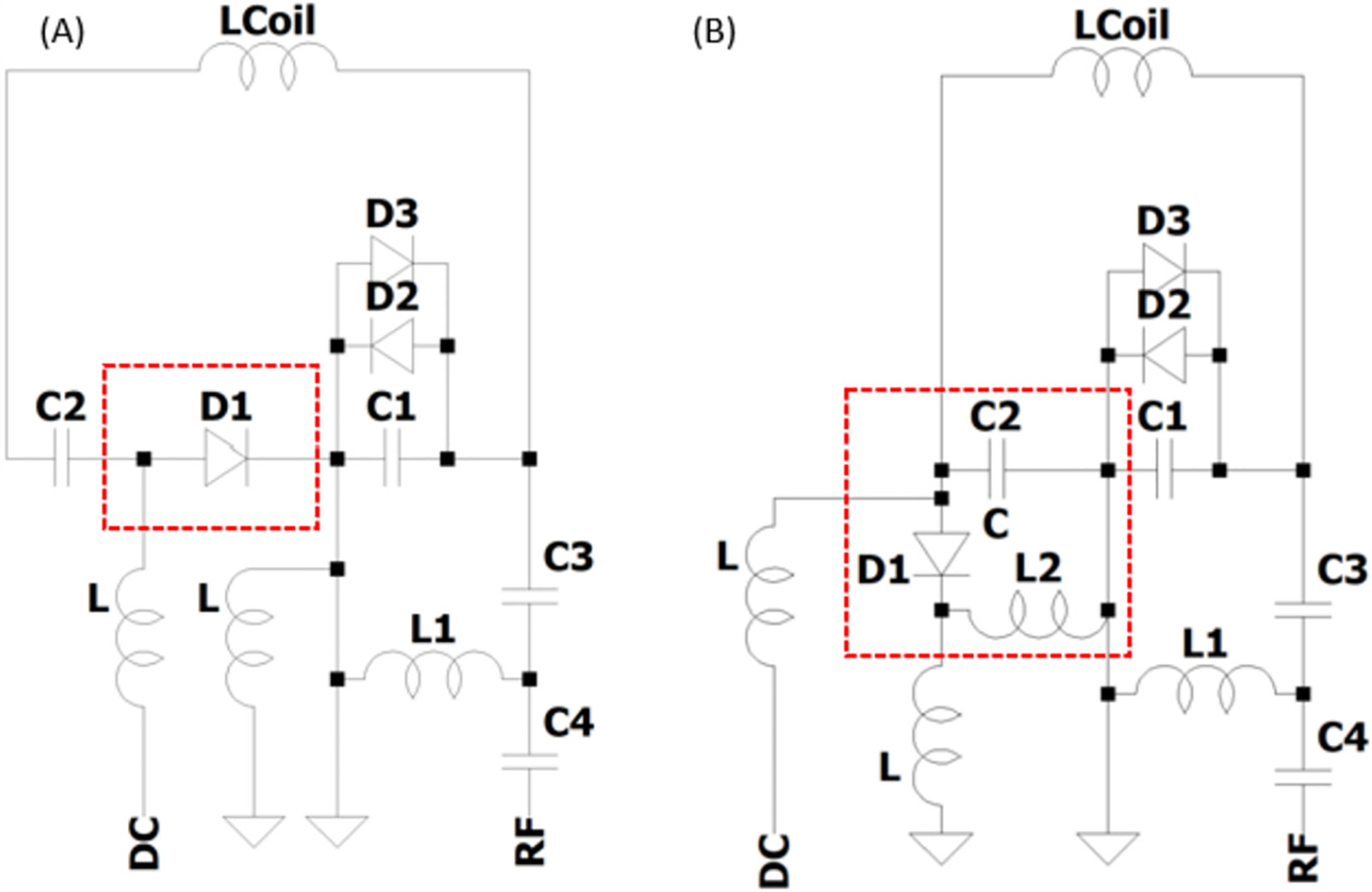
Schematic of coil match/tune circuitry and preamp interface (A) Broadband decoupling circuit using a PIN diode in series with the coil. (B) Reference circuit using a narrowband trap. D1: active switching diode, D2/D3: back-to-back diodes for preamp protection, L: RF chokes for DC lines, C1/C2: Tuning capacitors, C3/C4/L1: matching/impedance transformer network, L2: decoupling trap inductance, LCoil: coil inductance.

To create modular triple-tuned coils, Tx and Rx coils tuned to ^23^Na (32.59 MHz), ^31^P (49.87 MHz) and ^1^H (123.2 MHz) at 3 T were developed (Table 1). A set (coil set 1 in Table 1) of three single-tuned single-channel Rx coils (tuned to each frequency) were designed using a conventional narrowband trap for active detuning as is reported in the literature for single-tuned coils [18]. This was compared to a set of three single-channel coils tuned to each frequency that implemented a series PIN diode to demonstrate broadband decoupling (coil set 2 shown in Table 1). The coils were designed on a custom former that enabled stacking of the coils. Each coil consisted of a 7 cm loop following the circuit schematics in Fig. 1 using 18 AWG insulated wire (Belden), fixed capacitors (Passive Plus, 1111C series), variable capacitors (Johanson, 9702 Series), inductors (Coilcraft Inc., 1812 Series), and PIN diodes (Macom MAP7470). Coil ports were offset by 120° to maximize separation between match/tune circuitry for each element. Match/tune circuitry and the integrated preamplifier networks were milled on 1 oz. copper clad FR4. Low input impedance preamplifiers (WanTcom Inc., WMM Series) were used to achieve the required gain for the Siemens (3 T Verio) system. Although preamplifier decoupling was not necessary for the single-channel coils used in this experiment, it enables further modularity in future studies using these coils to increase the field of view (FOV) and sensitivity through implementation of multi-nuclear phased arrays. Finally, a single-tuned three-element planar ^1^H array (coil set 3 in Table 1) was constructed using the broadband decoupling network (Fig. 1A) to further demonstrate the positioning independence of the decoupling. This array was also useful to highlight its applicability to more complex multi-nuclear array coils necessary for widespread clinical utility. To evaluate the Rx coils on the bench, coils were characterized for matching (S_11_), Q, and inter-element coupling (S_21_) for the array. Preamplifier decoupling and active detuning were measured with a decoupled probe.

Similarly, two Tx-only butterfly coils were constructed: a double-tuned ^1^H–^31^_P_ coil and a single-tuned ^23^Na coil (coil set 4 in Table 1). The butterfly coils were designed to sit on top of the Rx arrays on the coil former with dimensions optimized for roughly 9 cm depth of sensitivity as outlined by Kumar et al. [19]. This depth was selected to correspond to the rectus femoris muscle in large animal models with the thickness of the final stacked coil configuration taken into account. The butterfly coils were coarsely tuned with distributed capacitors (Passive Plus, 1111C series) and finely tuned with variable capacitors (Voltronics, NMAT 40HVE). PIN diodes (MACOM, MA4P7470) were used to actively tune the butterfly coils. RF chokes (Coilcraft Inc., 1812Cs Series) were placed in parallel with the distributed tuning capacitors. A bias tee was constructed to combine the DC PIN diode drivers from the Siemens interface into a single port on the coil. The double-tuned butterfly was tuned using the pole insertion method by replacing all the distributed fixed capacitors with LCC traps [7].

### 2.2. Scanner SNR comparisons

**Fig. 2.**
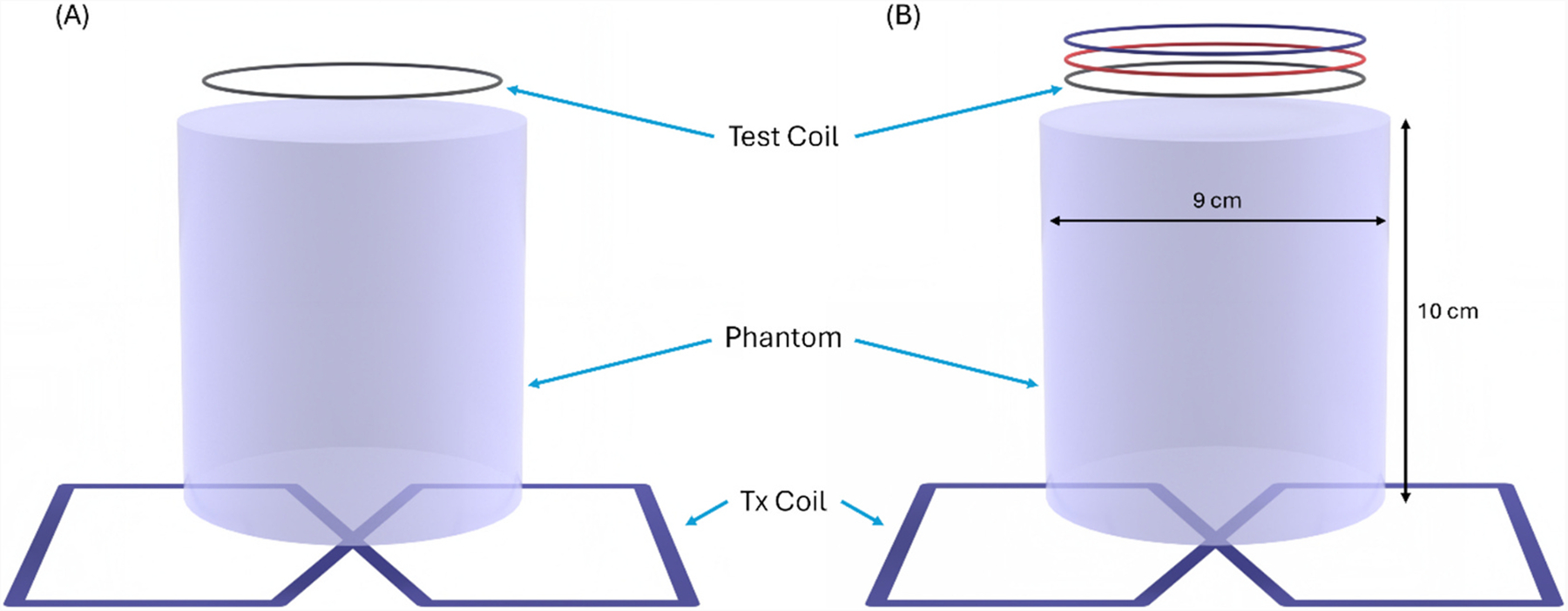
Diagram of the coils on the phantom with the Rx coils in the (A) unstacked and (B) stacked configurations.

SNR comparisons were performed on a 3 T Siemens Verio scanner. To characterize the coils in the scanner, a test platform was constructed with 3/16 in. diameter guide rods and spacers to allow stacking of the various coil configurations and combinations in a consistent position between tests. Two homogeneous phantoms were made with 2 % agar, 100 mM of potassium phosphate, and 85 mM of NaCl to provide high sensitivity and load the coils. The phantoms were made in cylindrical glass jars with a 9 cm diameter and 11 cm height. The phantom was placed in between the Tx and Rx coils to enable stacking comparisons without moving the setup, as shown in Fig. 2.

The test platform was designed to fit into indents in the patient table for coil positioning to ensure it locked into the same location between coil setups. Acrylic cutouts were added to the setup to center the phantoms concentrically and evenly space them between coils every time configurations were changed. Crosshairs were etched into the acrylic to ensure consistent positioning of the test platform with the Siemens laser positioning system. For ^1^H comparisons, coronal slices were taken approximately 1 cm deep into the phantom to observe maximum potential coupling effects. For rigorous comparisons, parameters and ROI were selected following the recommendations of NessAiver for consistent SNR measurements [20]. A standard gradient echo (GRE) pulse sequence was used with the following parameters: repetition time (TR) = 200 ms, echo time (TE) = 20 ms, flip angle (FA) = 25°, matrix size = 256 × 256, FOV = 300 × 300 mm, slice thickness = 1.5 mm, bandwidth (BW) = 240 Hz/Px. Signal and noise regions were determined *via* script to mask the phantom. The signal region was defined as the entire phantom excluding the six voxels closest to the edge to eliminate any edge artifacts. Similarly, the rest of the image excluding the six voxels closest to the phantom were used for absolute quantification of the noise. This same experimental setup was used with both the broadband decoupling mechanism and the narrowband trap coil design to highlight the decoupling benefits of broadband decoupling for modular arrays. In both cases the 7 cm coils were stacked in alignment to generate the worst-case configuration for coupling. To ensure accurate comparisons between stacking configurations, the coil being used for reception remained on the bottom to maintain a consistent distance from the phantom. Finally, this was repeated with a three-element ^1^H array both independently and combined with the single-channel 7 cm X-nuclei coils (^23^Na and ^31^P) to demonstrate the utility to more complex array coils. For this test the slice positioning of approximately 4.5 cm into the phantom was used to mimic a realistic depth of muscle *in vivo*.

Similar comparisons were made for the X-nucleus coils to quantify the effects of the modular coil design. Bulk ^31^P and ^23^Na spectroscopy data were acquired for SNR comparisons. ^31^P spectra were acquired with: TR = 6000 ms, TE = 0.35 ms, pulse duration = 500 μs, FA = 90°, no. points = 1024, BW = 1000 Hz, no. averages = 16. Similarly, ^23^Na spectra were acquired with: TR = 1050 ms, TE = 0.35 ms, pulse duration = 500 μs, FA = 90°, no. points = 1024, BW = 1000 Hz, no. averages = 16. Spectra were processed using McMRSGUI, an open-source GUI developed in house [21,22]. Averaging, 0 and 1st order phasing were performed on the data. For multi-channel datasets, data from each channel was averaged and phased individually prior to channel combination.

To verify the broadband design could be used for the acquisition of physiologically relevant signals, data was acquired on a pig immediately post-mortem. The pig was placed in a right lateral recumbent position to enable access to the leg muscles. The ^1^H array and the two 7 cm X-nuclei coils were stacked and placed on the hip. A localizer sequence was used to identify the bicep femoris muscle. The multinuclear protocol included a ^1^H T_1_ weighted (T_1_w) GRE and due to sequence availability, bulk spectroscopy for ^31^P and ^23^Na. The T_1_w GRE parameters were as follows: TR = 700 ms, TE = 8.9 ms, FA = 90°, matrix size = 256 × 256, FOV = 185 × 185 mm, slice thickness = 5 mm, BW = 260 Hz/Px. The ^31^P spectroscopy parameters were TR = 2080 ms, FA = 90°, matrix size = 2048, BW = 3000 Hz, no. averages = 128. The bulk ^23^Na spectroscopy parameters were TR = 1060 ms, FA = 90°, matrix size = 1024, BW = 2000 Hz, no. averages = 128. This study was approved under Texas A&M University’s Institute for animal use and care committee under protocol number IACUC 2024–0047.

## 3. Results

### 3.1. Coil design and construction

**Fig. 3.**
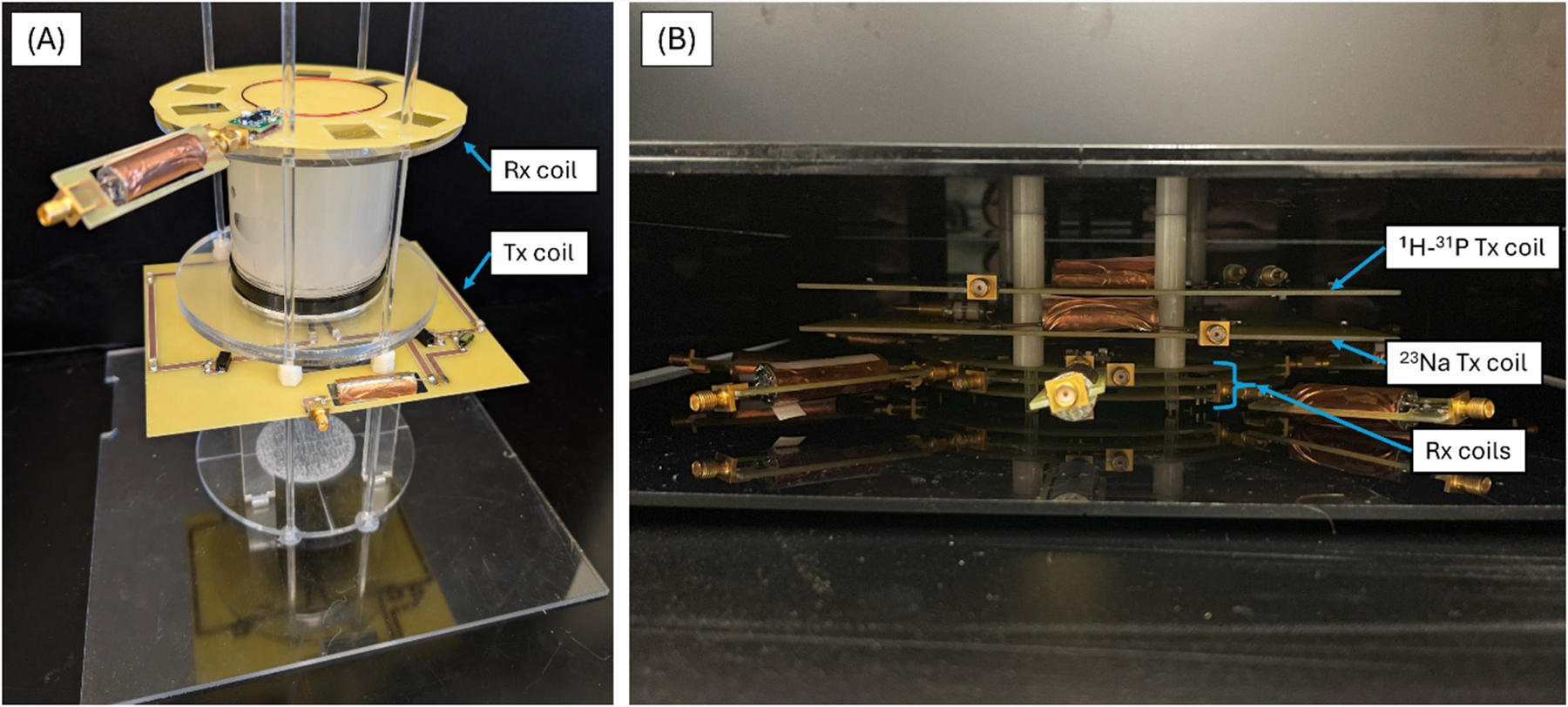
Photographs of coils (A) The test platform used for stacking comparisons. The Tx butterfly coil is on the bottom with a phantom on top. A single representative Rx coil is placed on top of the phantom with the rails available for stacking additional coils on top. (B) The coils positioned in the final box setup to be used for post-mortem studies. This configuration was designed for modularity such that Rx elements could be interchanged to tailor to a given study. The Rx coils should be placed closest to the imaging subject to achieve the highest reception sensitivity, in this case the coil box would be placed on top of the subject. Receive coils are positioned from top to bottom: ^1^H, ^31^P, ^23^Na as shown.

Photographs of the constructed test platform (Fig. 3A) show a single Tx coil below a phantom with a single Rx coil stacked on top. This test platform allowed for the stacking of different Rx combinations without moving the Tx coil or phantom for consistent comparisons between sets. This test platform allowed for the characterization of coils before combining them into a single coil box (Fig. 3B) for *in vivo* studies. This setup consisted of three Rx coils (one for each frequency) and two Tx coils (double-tuned.^1^ Coil bench metrics are summarized in Table 2. All coils were matched to at least −19.6 dB. The broadband design showed a significant drop in both Q_unloaded_ and the Q ratios as compared to the narrowband trap network. Although this drop is significant, it is to be expected as the PIN diodes have a significant series resistance which leads to higher insertion loss than the narrowband traps. Nonetheless, in all cases, this improved the active detuning of the coil compared to the trap design.

### 3.2. Imaging and spectroscopy studies

**Fig. 4.**
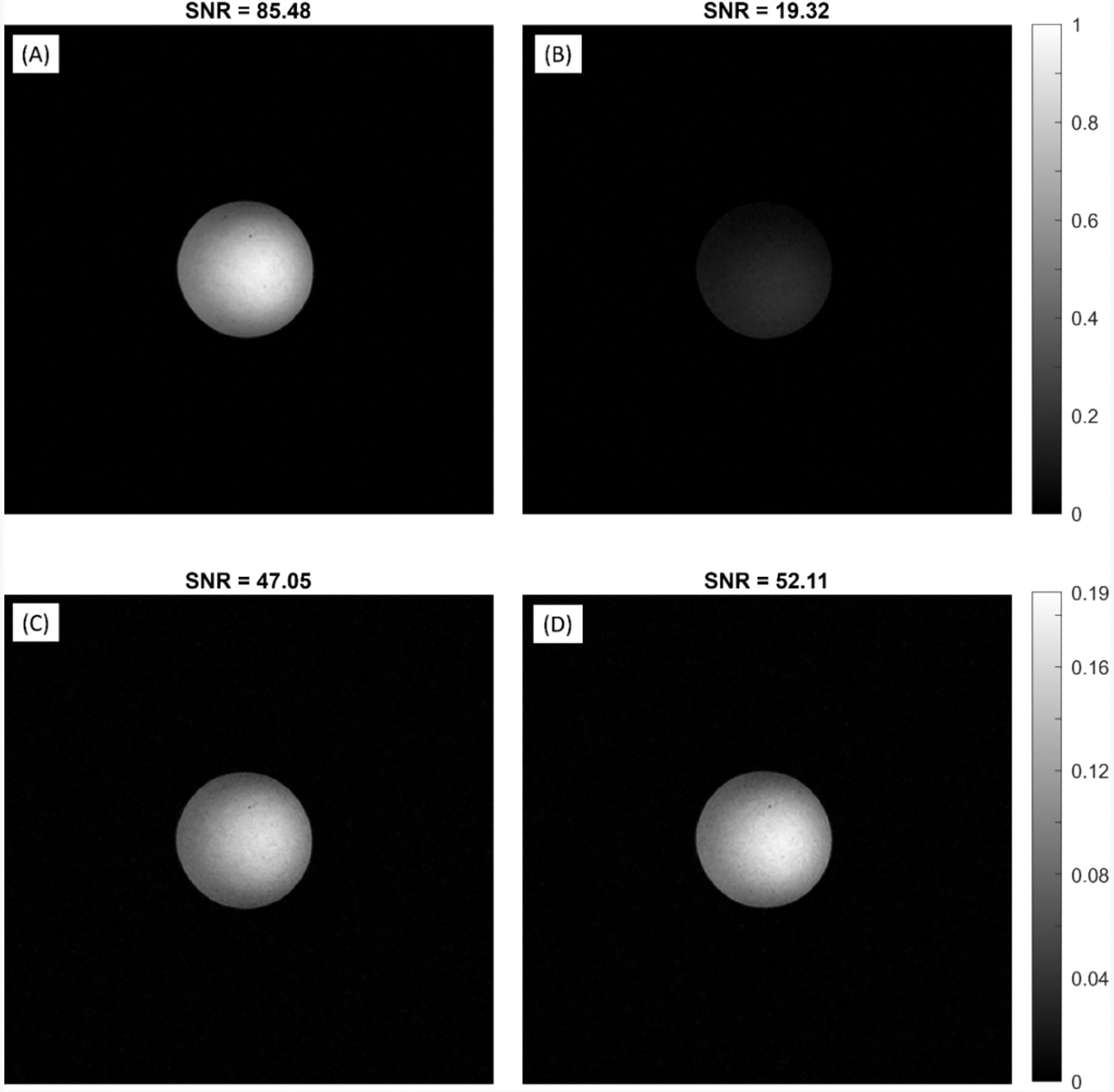
^1^H reference images. SNR calculated *via* the NessAvair method [20]. (A) A single narrowband trapped ^1^H coil. (B) Stacked narrowband trapped ^1^H, ^31^P and ^23^Na coils. (C) A single broadband decoupled ^1^H coil. (D) Stacked broadband decoupled ^1^H, ^31^P and ^23^Na coils.

As a reference, the single channel scanner measurements of ^1^H with the standard narrowband trap showed the expected highest SNR (85.48) (Fig. 4A). However, this design, when stacked with the three narrowband trap detuned X-nucleus coils (Fig. 4B), showed an expected and notable drop in SNR to 19.45 presumably due to coupling between the Rx coils (despite being tuned to different frequencies and with active detuning traps) due to the inherent narrowband nature of these traps. This coupling is difficult to experimentally measure due to the unmatched nature of measurements between coils tuned to different frequencies. Conventional S_21_ coupling measurements suggest misleading, and in certain cases inaccurate, conclusions regarding inter-element coupling. For the broadband decoupling case, the SNR did not change significantly (47.05 *vs* 52.11) between the unstacked (Fig. 4C) and stacked (Fig. 4D) configurations, respectively, indicating the relative immunity to coupling this network provided using a more straightforward and miniature decoupling circuit.

X-nuclei comparisons using bulk spectroscopy (Table 3) showed similar trends to those shown with ^1^H imaging. Stacking of the ^31^P coil using narrowband detuning traps resulted in coupling which prevented re-tuning of the coil prior to data acquisition. A spectrum was acquired, but no signal peak could be identified for SNR calculations. Alternatively, with the broadband decoupling network the SNR varied by roughly 5 % and the coils could easily be tuned to their respective resonant frequencies. ^23^Na spectra showed a drop in SNR of roughly 50 % in the narrowband design due to coupling. When these coils were replaced by broadband decoupled coils, the SNR was stable between the stacked and unstacked configurations with a variation of roughly 7 %.

**Fig. 5.**
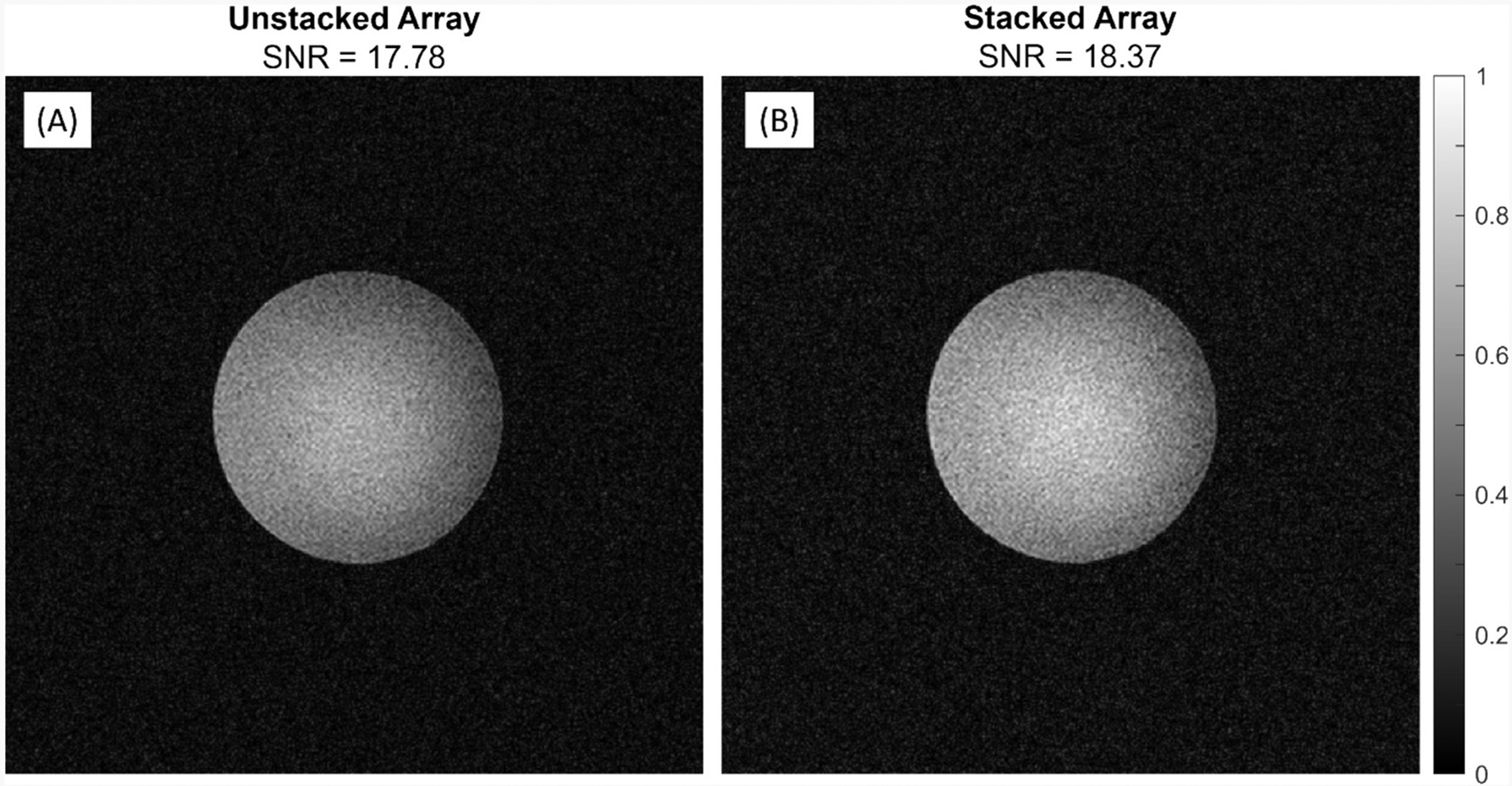
(A) A three element single-tuned ^1^H array with a broadband decoupling network. Coronal slice taken at 4.5 cm into the phantom to mimic a deep skeletal muscle slice. (B) Single-channel ^31^P and ^23^Na broadband decoupled coils were stacked on top to show no significant change in SNR as a result of coupling.

Images acquired with the ^1^H array unstacked (Fig. 5A) and stacked (Fig. 5B) with the two 7 cm X-nuclei coils showed that the SNR between the two combinations again did not change significantly (less than 4 %). The slice depth was changed to 4.5 cm to evaluate performance of the array deeper into the phantom as it would be, presumably, *in vivo* which led to a lower SNR than seen with the single-channel coils.

**Fig. 6.**
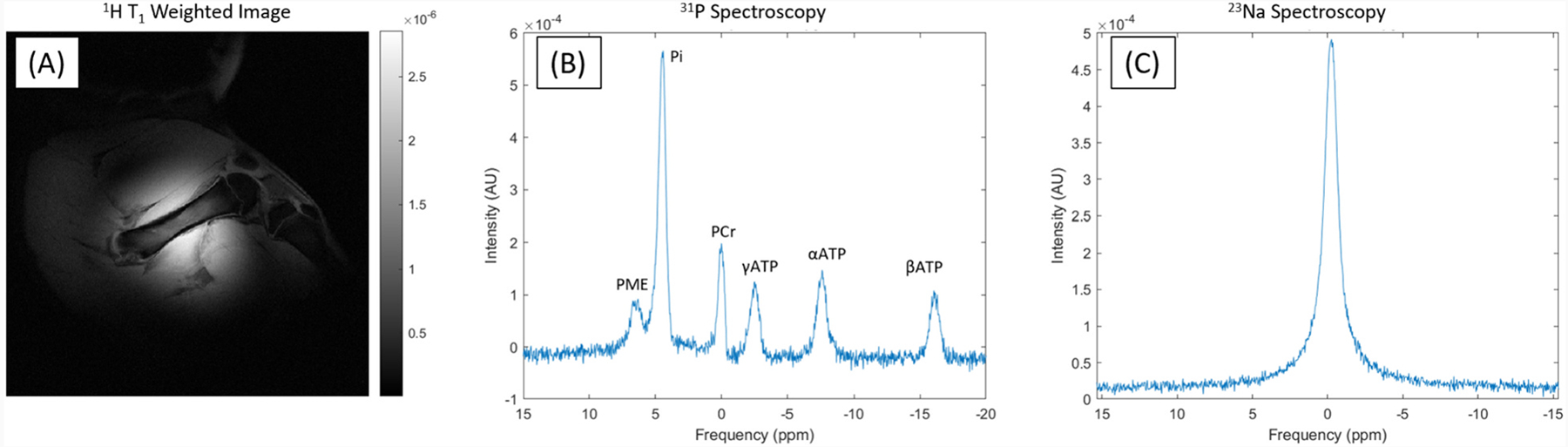
Post-mortem imaging: (A) ^1^H images showing high resolution in a porcine model. (B) Bulk ^31^P spectra showing sufficient SNR to see all three phosphate groups of ATP, Pi, PME, and PCr. (C) Bulk ^23^Na spectra showing sufficient SNR at physiologic concentrations.

A ^1^H T_1_w image (Fig. 6A) showed sub-millimeter resolution images over the hip of the pig, with no visible distortion due to coil coupling. Bulk ^31^P spectra (Fig. 6B) were acquired over the same region. In these spectra the expected metabolites were observed including phosphomonoesters (PME), inorganic phosphate (Pi), phosphocreatine (PCr), and the γ, α, and β phosphate groups of adenosine-triphosphate (ATP) in agreement with published findings on post-mortem pig studies [23]. Although there is no chemical shift with bulk ^23^Na spectroscopy (Fig. 6C), the spectrum shows sufficient SNR at physiologically relevant concentrations for coil validation.

## 4. Discussion

The broadband decoupling network enabled modular recombination of surface coils to create an effectively triple-tuned coil system. Data presented here shows that with the broadband design SNR measurements were stable with different combinations of coil configurations as compared to narrowband trap circuits which showed significant signs of inter-element coupling. The stacked coil configuration, representing almost perfectly geometrically coupled coils, and the operational frequencies serve as proof of concept to highlight the frequency and position independence of the proposed decoupling mechanism. Furthermore, future developments in biological studies using these coils may reveal interest in additional biomarkers that might benefit from additional nuclei. The broadband nature of this design would enable the combination of relatively simple single-tuned hardware following this broadband schematic to allow for sensitivity to additional nuclei without the need for custom multi-nuclear coil designs. This presents a significant benefit of the design for biomarker development studies where the biological significance of specific nuclei is yet to be fully realized.

Both benchtop and scanner data show significant reduction in sensitivity as a result of the broadband decoupling mechanism. In particular for ^23^Na spectra, the SNR decrease from the broadband decoupling was comparable to that of the coupled coils. With this design, however, signal integrity is retained from unstacked *versus* stacked comparisons. While there may not be an inherent SNR benefit to this design in its current form at all frequencies, it ensures that the signal measured is a stable and accurate metric of the signal originating from the sample volume, not one artificially skewed by inter-element coupling.

This loss in sensitivity could also be improved with the implementation of PIN diodes with a lower series resistance. PIN diodes are currently readily available on the market with series resistances as low as 0.2 Ohms (a 75 % reduction in resistance) which would generally expect an SNR improvement inversely proportional to the square root of the change in resistance [24]. These low loss PIN diodes, however, were not available in a non-magnetic package and have many constraints that must be considered in the selection of appropriate PIN diodes [25]. PIN diode series resistance is typically inversely proportional to bias current. An increase in bias current could help to improve the SNR of the coils presented here but typically is constrained by manufacturer limits. Additional constraints that affect PIN diode selection include the off-state capacitance. A sufficiently low capacitance is necessary to ensure effective off-state isolation to fully decouple the coils. Higher capacitances may allow coupling even for a reverse biased diode, an effect that only worsens at higher frequencies associated with high field studies. Reverse bias leakage current, must also be minimized to ensure sufficient isolation in the decoupled state. Similarly, bias conditions must be matched to the available system outputs to ensure proper switching performance of the coil and minimize potential noise sources. Diode switching speeds must also be considered and has been shown to be limiting particularly for passive switching applications at low fields [26]. All of these considerations must be taken into account when selecting the appropriate PIN diode for this application.

While the SNR losses shown here are significant, the position independence of the decoupling mechanism would enable future incorporation of phased arrays to mitigate the effects of these losses while providing design flexibility for multinuclear coils. While it is suggested in most instances that 70 % of the ^1^H SNR and 90 % of the second-nucleus SNR be maintained in comparison to single-tuned counterparts [10], the practical applicability of a coil in terms of SNR requires a more holistic evaluation of both coil design and the needs of a given study. Implementation of phased arrays and optimization of preamplifier circuitry can both be used to significantly improve the SNR of the resultant coil system in some cases. Stacked coil configurations have been reported in the literature to generate homogeneous fields at low field strengths [27]. This particular stacked coil configuration targets broadband (multi-tuned) applications. In terms of application, high concentrations close to the surface such as the application of this study may still allow evaluation of biomarkers of interest as shown in Fig. 6. This implementation of a phased array was shown for ^1^H to demonstrate the proof of concept in terms of coupling but could be implemented for X-nucleus arrays as well. Additionally, the inclusion of preamplifier decoupling would enable combinations with more coils to increase the field of view or depth of sensitivity of the coil in future iterations of the design with little to no changes being required for the original coil setup. This could lead to significant cost savings in the design and implementation of multi-nuclear array coils. Future work will include further investigation into the sensitivity benefits of modular planar arrays using this design. Specifically, the design in its current form and tuned to these particular frequencies is applicable for the study of biomarkers of musculoskeletal disease in large animal models (porcine and canine).

## 5. Conclusion

The design presented here enabled a straightforward decoupling mechanism that is independent of both frequency and position for the stacking of coils tuned to different frequencies. The data presented show how this decoupling mechanism can be used to create a modular triple-tuned coil system with either single-channel or array coils. While the coil losses were dominated by the PIN diode, the method allowed for the stacking of multiple coils tuned to different frequencies, with minimal coupling effects. Future work will entail investigation of X-nuclei array applications and musculoskeletal biomarkers in animal models using these coils.

## Figures and Tables

**Table 1:** Coil Configurations.

Coil set #	Coil description	Nucleus	Coil purpose
1	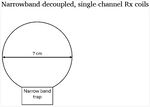	^1^H ^23^Na ^31^P	These three coils served as reference coils using narrowband traps (Fig. 1B) to compare to the broadband decoupling network.
2	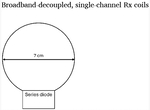	^1^H ^23^Na ^31^P	These three coils were developed to demonstrate the performance of the broadband decoupling circuit described in this work using the series PIN diode (Fig. 1A).
3	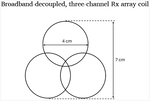	^1^H	This array served to show that more complex coils still decouple using the broadband mechanism (Fig. 1A).
4	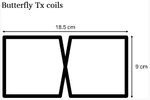	^1^H- ^31^P ^23^Na	These two coils were used as a Tx-only coil set composed of a double-tuned ^1^H—^31^P coil and single-tuned ^23^Na coil.

**Table 2:** Coil Bench Measurements.

Nucleus	Coil	S_11_ (Unitless)	Q_U_ (Unitless)	Q _Ratio_ (Unitless)	Preamp decoupling (dB)	Active detuning (dB)
^1^H	Tx	−38.2	81.2	1.05	N/A	N/A
	Rx Array, Ch 1	−27	76.8	1.43	−21.4	−20.8
	Rx Array, Ch 2	−28.9	65.2	1.12	−21.7	−18.3
	Rx Array, Ch 3	−23.5	62.8	1.23	−20.8	−17.9
	Broadband Rx	−21.9	92.8	1.91	−16.4	−25.1
	Narrowband Rx	−23.6	136.7	2.56	−12.2	−13.4
^31^P	Tx	−15	36.2	1.00	N/A	N/A
	Broadband Rx	−36.5	80.7	1.28	−16.5	−40.5
	Narrowband Rx	−24	210.1	1.94	−12.5	−9.4
^23^Na	Tx	−30.8	31.8	1.00	N/A	N/A
	Broadband Rx	−30.9	54.9	1.39	−17.2	−26.6
	Narrowband Rx	−19.6	177.4	1.49	−17.1	−15.1

**Table 3:** Stacked SNR Comparisons.

Receive nucleus	Configuration	Unstacked (AU)	Stacked (AU)
^1^H	Narrowband	85.5	19.3
	Broadband	47.1	52.1
^31^P	Narrowband	22.2	N/A*
	Broadband	17.1	16.2
^23^Na	Narrowband	35.0	17.0
	Broadband	20.8	19.2

* Stacking of the ^31^P coil with narrowband traps resulted in coupling which prevented retuning of the coil prior to data acquisition which artificially reduced the SNR below the measurable threshold.

## Data Availability

Data will be made available on request.
